# The relationship between pre-incarceration related social determinants and glycaemic control in African Americans with criminal legal involvement and type 2 diabetes

**DOI:** 10.1111/dme.70342

**Published:** 2026-04-26

**Authors:** Olaitan Akinboboye, Rebekah J. Walker, Jennifer A. Campbell, Laura Hawks, Joni S. Williams, Leonard E. Egede

**Affiliations:** 1 Department of Family and Community Medicine, Homer Stryker M.D. School of Medicine, Western Michigan University, Kalamazoo, Michigan, USA; 2 Division of Population Health, Department of Medicine, Jacobs School of Medicine and Biomedical Sciences, University at Buffalo, Buffalo, New York, USA; 3 Division of General Internal Medicine, Department of Medicine, Medical College of Wisconsin, Milwaukee, Wisconsin, USA; 4 Center for Advancing Population Science (CAPS), Medical College of Wisconsin, Milwaukee, Wisconsin, USA

**Keywords:** glycaemic control, pre-incarceration, social determinants, type 2 diabetes

## Abstract

**Aims:**

To evaluate the relationship between pre-incarceration social determinants, demographic and clinical factors, and glycaemic control in African Americans with a history of criminal legal involvement and type 2 diabetes mellitus.

**Methods:**

One hundred and seventy-one adults were recruited from medical records and community centres in the Midwestern United States. The model was estimated using stepwise regression with forward selection to evaluate the relationship between pre-incarceration factors, demographic factors, clinical factors and glycaemic control among African Americans with a history of criminal legal involvement and type 2 diabetes.

**Results:**

The fully adjusted model showed that age, comorbidity count and medication were significantly associated with glycaemic control. Compared to using only oral hypoglycaemic agents, the combination of pills/oral hypoglycaemic agents and insulin (*β*: 1.30; 95% CI: 0.60, 1.99) and using insulin or injectables alone (*β*: 0.87; 95% CI: 0.07, 1.67) were associated with an increase in HbA1c. Additionally, each additional comorbidity (*β*: −0.22; 95% CI: −0.37, −0.08) was associated with a 0.22% decrease in HbA1c, and each unit increase in age (*β*: −0.05; 95% CI: −0.09, −0.01) was associated with a 0.05% decrease in HbA1c.

**Conclusion:**

Pre-incarceration factors were not associated with glycaemic control in African Americans with a history of criminal legal involvement and type 2 diabetes. Interventions that account for factors such as comorbidity, age and medication are essential for future research.

## 1 | INTRODUCTION

Approximately 38.4 million people, or 11.6% of the US population, live with diabetes.^1^ In 2017, the economic impact of diagnosed diabetes in the United States was $413 billion, a 41% increase from the 2012 estimate of $245 billion.^1,2^ Those with diagnosed diabetes had medical expenses 2.3 times higher than those without diabetes.^1^ Over 60% of these costs were attributed to prescription drugs for blood glucose control and hospitalizations due to diabetes-related complications.^3^ Racial and ethnic disparities exist in diabetes-related healthcare expenses; for example, per-capita healthcare expenditures are higher for African Americans with diabetes ($10,470) compared to non-Hispanic whites with diabetes ($9800).^1^ While glycaemic control differences contribute to these disparities, research also indicates significant contributions from social determinants of health.^4,5^

The social determinants of health (SDOH) encompass non-medical factors that impact health outcomes.^6^ They are the conditions in which people are born, grow, work, live and age, and the wider set of forces and systems shaping the conditions of daily life.^6^ These determinants significantly impact health outcomes, as evidenced by studies indicating that approximately 30%–55% of such outcomes can be attributed to SDOH.^6^ Moreover, recent research has unveiled an independent association between SDOH and glycaemic control.^5,7,8^

Social determinants impact everyone, extending beyond marginalized individuals.^9^ On the other hand, social risk factors encompass adverse circumstances at the individual or family level that are associated with poor health outcomes and can amplify health disparities.^9^ Incarceration, a social risk factor, adversely affects economic opportunities, education, social support and housing security, influencing well-being and health outcomes for individuals and communities.^10^ While there is significant attention given to the connection between the criminal legal system and health outcomes,^11,12^ evidence suggests that certain factors preceding criminal legal involvement, such as locus of control (LOC) and adverse childhood experience (ACE), impact health, including glycaemic control in individuals with diabetes.^13–17^ Rotter introduced the concept of LOC to explain how individuals perceive the link between their actions and outcomes.^18^ While research has shown a negative relationship between LOC and self care in diabetes, no direct association was found with glycaemic control.^13^ ACE refers to traumatic events before the age of 18,^19^ which disrupt health, opportunities and stability, influencing future generations.^19^ Notably, individuals with type 2 diabetes and ACE often exhibit poorer glycaemic control,^14^ with racial and ethnic disparities in adversity exposure, such as 61% of African American children experiencing at least one ACE compared to 40% of non-Hispanic white children.^20^

Limited research has explored the health of justice-involved African Americans, especially regarding non-communicable chronic illnesses.^21^ This study aims to investigate the relationship between pre-incarceration factors and glycaemic control in African Americans with type 2 diabetes mellitus (T2DM), hypothesizing that pre-incarceration factors such as ACE and low LOC before the initial criminal legal involvement post-18 years will be associated with poor glycaemic control.

## 2 | METHODS

### 2.1 | Study design, sample and setting

This cross-sectional study included a sample of 171 African American individuals with T2DM who had a history of criminal legal involvement. Participants were recruited from various sources, including hospital medical records, community sites such as places of worship, clinics, homeless shelters, local food pantries, community events and referrals from enrolled participants, re-entry organizations and community leaders in the greater Milwaukee area in the Midwest region of the United States. To be eligible for participation, individuals had to be 18 years of age or older, identify themselves as Black/African American, report a clinical diagnosis of T2DM, indicate a history of contact with the justice system and be able to communicate in English. Participants were excluded from the study if they displayed signs of mental confusion during the screening assessment, reported an active psychosis or acute mental disorder, or were currently under house arrest or continuous electronic monitoring. Prior to the commencement of the study, all participants provided written informed consent prior to enrollment. Measures were taken to ensure confidentiality, voluntary participation and protection against coercion or undue influence. We obtained necessary approvals from the institutional review board at the Medical College of Wisconsin.

## 3 | DATA COLLECTION

After we received IRB approval, a review of the electronic medical records was conducted to identify participants who were eligible for the study. Once identified, we contacted these eligible participants via phone and provided them with a detailed description of the study. Additionally, we approached participants directly in clinics, community events and re-entry organizations. Those who expressed interest and met the eligibility criteria were then consented and given a questionnaire to complete. The questionnaire covered various socio-demographic factors, as well as pre-incarceration factors. Blood samples were collected from all participants in order to measure the outcome variable, HbA1c.

## 4 | CONCEPTUAL FRAMEWORK

The conceptual framework of this paper draws upon the Life Course Theory, which acknowledges the interplay between an individual, their environment and the passage of time.^22^ Life course perspectives illustrate that an individual’s present health is influenced by past exposures, often spanning decades, to physical, environmental and psychological factors.^22^ The life course approach encompasses at least two predominant perspectives—developmental and structural—each offering distinct yet complementary theories and methodologies that can be utilized to tackle the issue of health disparities.^22^ Timing of life events, including past events such as ACE, and human agency and personal control, such as locus of control, can be related to health outcomes^19,23^ (See Figure 1). For this analysis, we hypothesized that pre-incarceration factors such as ACE and LOC prior to the first criminal legal involvement after the age of 18 years will be significantly associated with poor glycaemic control after controlling for relevant covariates.

## 5 | OUTCOME MEASURES

Haemoglobin A1c (HbA1c) was the outcome measure. A trained phlebotomist collected blood samples, which were then sent to the Wisconsin Diagnostic Laboratory (WDL) for processing. HbA1c was treated as a continuous variable.

## 6 | PRE-INCARCERATION FACTORS

### 6.1 | Locus of control

The Brief LOC scale, as utilized in our study, evaluated participants’ locus of control. This scale employed a five-point Likert scale format, as suggested by Levenson (1974), incorporating six items based on Rotter’s Internal-External LOC Scale.^24^ Previous data support the scale’s predictive validity and indicate it as a practical alternative when the full scale is not feasible. Each of the six questions required participants to rate their agreement on a scale ranging from 1 (strongly disagree) to 5 (strongly agree).^24^ The scale encompasses three items assessing the internal LOC dimension and an additional three items examining the external LOC.^24^ Scores on the five-point Likert scale are interpreted such that higher values suggest a higher internal or external LOC.^24^ Individuals with an internal locus of control believe that their health status is primarily influenced by their own inherent ability to manage their health condition.^25^ Conversely, those with an external LOC hold the belief that their health is determined by powerful external forces or by chance.^26^ The Brief LOC scale exhibits a good internal consistency, as indicated by a Cronbach’s alpha coefficient of 0.68.^24^

### 6.2 | Adverse childhood experience (ACE)

The ACE module comprises a 17-item survey that captures various adverse events occurring in the respondents’ childhood, specifically before the age of 17. Out of these, 14 items were utilized to categorize adverse childhood experiences into 14 categories: sexual abuse (3 items), emotional abuse (1 item), physical abuse (1 item), emotional negligence (1 item), physical negligence (1 item), domestic violence (1 item), substance abuse (1 item), mental illness (1 item), incarceration (1 item), witnessed violence (1 item), witnessed discrimination (1 item), adverse neighbourhood (1 item), bullied (1 item), ever lived in a foster care (1 item). For questions with ‘yes’ or ‘no’ response options, an ACE was endorsed if they answered yes to the question. Whereas, for questions with response options of once, a few times or no; once or a few times were collapsed into one category and used to indicate exposure to the specific ACE. The ACE categories were then grouped into conventional and expanded ACE. The conventional ACE score included emotional abuse, physical abuse, sexual abuse, emotional negligence, physical negligence, witnessing domestic violence, having a family member who struggled with substance abuse, mental illness and incarceration. The expanded ACE score encompassed witnessing violence, discrimination, adverse neighbourhood, being bullied and ever having lived in foster care.

## 7 | DEMOGRAPHIC AND COMORBIDITY MEASURES

Additional patient characteristics collected include age, gender, education, income, employment status, marital status, BMI, duration of diabetes, medical comorbidity and medication type. Age was analysed as a continuous variable. Gender was categorized as either men or women. Education was defined as the years of education beyond kindergarten and was analysed as a continuous variable. Annual income was dichotomized into < $25,000 and ≥ $25,000. Employment status was categorized as not employed or employed. Marital status was categorized as never married vs. married/separated/divorced/widowed. BMI was computed by dividing the respondent’s weight in kilograms by the square of their height in meters and was treated as a continuous variable. Diabetes duration was computed by subtracting the age at diabetes diagnosis from the current age of the participant, and it was also treated as a continuous variable. Medical comorbidities included hypertension, hyperlipidaemia, myocardial infarction, angina, stroke, asthma, cancer, COPD, connective tissue disease, depression, kidney disease, eye disease, end-stage renal disease and leg amputation. Medical comorbidities were self-reported with response options: ‘yes,’ ‘no’ or ‘I don’t know’. Each comorbidity was dichotomized, with response type ‘yes’ categorized as ‘yes’ and ‘no’ and ‘I don’t know’ categorized as ‘no’. Comorbidity count was generated as a total of endorsed comorbidities and was treated as a continuous variable. The type of diabetes medication was categorized as oral/pill, insulin, injectable medication/shot or a combination of these.

## 8 | STATISTICAL ANALYSES

After confirming the normal distribution of variables, mean ± SD or percentages were computed for demographic variables. Then, three sets of linear regression models were run to examine the relationship between pre-incarceration factors and glycaemic control, controlling for covariates using the conceptual model for the study (Figure 1). In the first set, unadjusted linear regression models were run with HbA1c as the outcome, and each pre-incarceration variable (LOC, expanded ACE and conventional ACE) as primary independent variables. In the second set, partially adjusted linear regression models were run with HbA1c as the outcome, and all three pre-incarceration variables (LOC, expanded ACE and conventional ACE) as primary independent variables in the same model. In the third set, stepwise linear regression models with forward selection were run with HbA1c as the outcome, and pre-incarceration variables (LOC, expanded ACE and conventional ACE) as primary independent variables, and age, gender, education, income, employment status, marital status, BMI, duration of diabetes, medical comorbidity and medication type as covariates. Entry into the model was based on a p-value of ≤0.2. Stepwise regression is frequently utilized for variable selection, especially in scenarios with numerous predictors and a single outcome, as seen in our study.^27^ All analyses were carried out using STATA Version 17 (Stata Corp LP, College Station, TX). A two-sided p-value of less than 0.05 was used to assess statistical significance.

## 9 | RESULTS

Figure 1 shows the conceptual model for the study. Table 1 shows sample characteristics of the 171 adults with prior criminal justice involvement and type 2 diabetes. The mean age was 57 years, with the majority being men (61%). A significant portion of the sample was unemployed (81%) and 88% earned an annual income of less than $25,000. The mean duration of diabetes was 17 years, and 44% used only oral medications. The average number of comorbidities was 4, and the average BMI was 35 kg/m^2^.

Table 2 shows the means and frequencies of both the primary independent and dependent variables. Within our study sample, the average HbA1c was 7.9% with a range of 4.4% to 14.1%. The mean score for LOC was 3.01 with a range of 1.17 to 4.50. Approximately 61% of participants reported experiencing physical abuse before the age of 18. Additionally, 17% stated that they endured emotional abuse during childhood and adolescence. In the sample population, 9.9% acknowledged experiencing emotional neglect, while 17.5% reported instances of physical neglect. A substantial number (62.6%) disclosed witnessing violence during their childhood and adolescence. Approximately 47% of the sample population disclosed experiencing domestic violence, and 51.5% reported having an adult in their home who struggled with substance abuse during their upbringing. Finally, 46.8% experienced exposure to four or more conventional ACEs, while 40.9% were exposed to three or more expanded ACEs.

Table 3 shows unadjusted, partially adjusted and fully adjusted stepwise linear regression with forward selection. In the unadjusted and partially adjusted models, there was no statistically significant relationship between pre-incarceration SDOH (locus of control, conventional and expanded ACE) and glycaemic control. In the stepwise regression, age, comorbidity count and medication were retained in the model and were statistically significantly associated with glycaemic control. Compared to pill or oral hypoglycaemic agent only, using both pill/oral hypoglycaemic agent and insulin (β-coefficient: 1.30; 95% CI: 0.60, 1.99) and using insulin or injectable only (β-coefficient: 0.87; 95% CI: 0.07, 1.67) were associated with an increase in HbA1c. Every additional increase in comorbidity count (β-coefficient: −0.22; 95% CI: −0.37, −0.08) was associated with a 0.22% decrease in HbA1c. Every unit increase in age (β-coefficient: −0.05; 95% CI: −0.09, −0.01) was associated with a 0.05% decrease in HbA1c. Employment status and expanded ACE scale were retained in the model but were not statistically significantly associated with glycaemic control.

## 10 | DISCUSSION

In this study of 171 African American adults with criminal legal involvement and type 2 diabetes, we found that pre-incarceration factors were not significantly associated with glycaemic control. Older age, having comorbidities and the type of medication used were significantly associated with glycaemic control, indicating that individuals in this sample who are older or have a higher number of comorbid conditions have better glycaemic control compared to those who are younger or do not have comorbid conditions. Additionally, individuals in this sample who use insulin only or insulin and oral diabetes medication have poorer glycaemic control compared to those who use oral diabetes medication only.

It is noteworthy that in our sample of African American adults with criminal legal involvement and type 2 diabetes, the prevalence of ACEs was relatively high compared to the general population^19,20^ and other populations with T2DM.^28^ For example, the prevalence of exposure to four or more conventional ACE in our study is 46.8%, which is significantly higher than the national prevalence of 17.3% reported for experiencing four or more types of ACEs in the United States^19,20^ and some studies have reported a much lower prevalence of 13%^15^ and 16.1%.^29^

Our study finding, showing that pre-incarceration factors such as ACE and LOC were not significant drivers of glycaemic control in our sample population, are consistent with prior studies of inner-city African American adults with T2DM.^30^ Additionally, our study findings show that older age, which is significantly associated with glycaemic control, are similar to current literature. For example, studies have consistently shown that older age is associated with better glycaemic control compared to younger age among minority populations with diabetes.^30,31^ The finding regarding comorbidity’s significant association with glycaemic control is mixed. Some studies showed that people with more comorbidity counts undergo more consultations and intensified treatment^32,33^; whereas other findings suggest that over time, the beneficial effect of comorbidity on glycaemic control diminishes.^34^ Additionally, our finding that the type of medication used is a significant driver of glycaemic control is consistent with the literature. Specifically, a study finding showed that the minority population who used insulin were less likely to achieve good glycaemic control.^35^ Also, studies have shown that the use of both insulin and oral diabetes medication is associated with a 2.7 times higher risk of poorer glycaemic control compared to oral diabetes monotherapy.^35^

To the best of our knowledge, this is one of the first studies to test the contribution of pre-incarceration factors to glycaemic control among African Americans with criminal legal involvement and type 2 diabetes using primary data collection of a comprehensive set of social determinants of health variables and clinical measures based on a well-researched conceptual framework. While SDOH are important, clinical factors are stronger drivers of glycaemic control in this population. Our study findings imply that efforts to optimize medications and increase medication adherence for this population may be the most important driver of good health outcomes, which is potentially linked to factors such as insurance coverage, access to care and aggressive medication titration by clinical providers.

Our study has several limitations. First, adverse childhood experiences were reported retrospectively by the respondents, potentially introducing recall bias and leading to underreporting of these experiences. Secondly, as the study was conducted in the Greater Milwaukee Area and data were collected at a single timepoint, it limits the generalizability of our study findings. Thirdly, our cross-sectional study demonstrates associations between certain socio-demographic factors and glycaemic control among African American adults with criminal legal involvement and type 2 diabetes mellitus; it cannot establish causality. Fourth, the study’s findings should be interpreted with caution due to the small sample size, which may limit the generalizability of the results to a larger population. Fifth, HbA1c was used as the primary outcome, but it can be influenced by conditions such as anaemia or kidney disease. Although comorbidities were considered, not all factors affecting HbA1c were adjusted for. Additionally, categorizing HbA1c may oversimplify glycaemic control, which is often individualized, potentially limiting applicability in heterogeneous populations. Future studies should consider both continuous and clinically stratified measures. Finally, this study measured two pre-incarceration SDOH. Future studies should include multiple factors that account for all domains of SDOH and leverage the use of a large sample size so as to have enough power to detect any difference. Despite these stated limitations, our study also boasts strength, as we employed a comprehensive set of validated survey measures to collect the information on social determinants of health.

## 11 | CONCLUSION

Pre-incarceration SDOH, measured as locus of control and adverse childhood experiences, were not associated with glycaemic control in African Americans with criminal justice involvement and type 2 diabetes in our study. However, comorbidity, age and type of anti-diabetes medication were associated with glycaemic control in this population. Therefore, strategies that take into account these influencing factors are warranted to achieve the desired glycaemic outcomes in our study population.

## Figures and Tables

**FIGURE 1:**
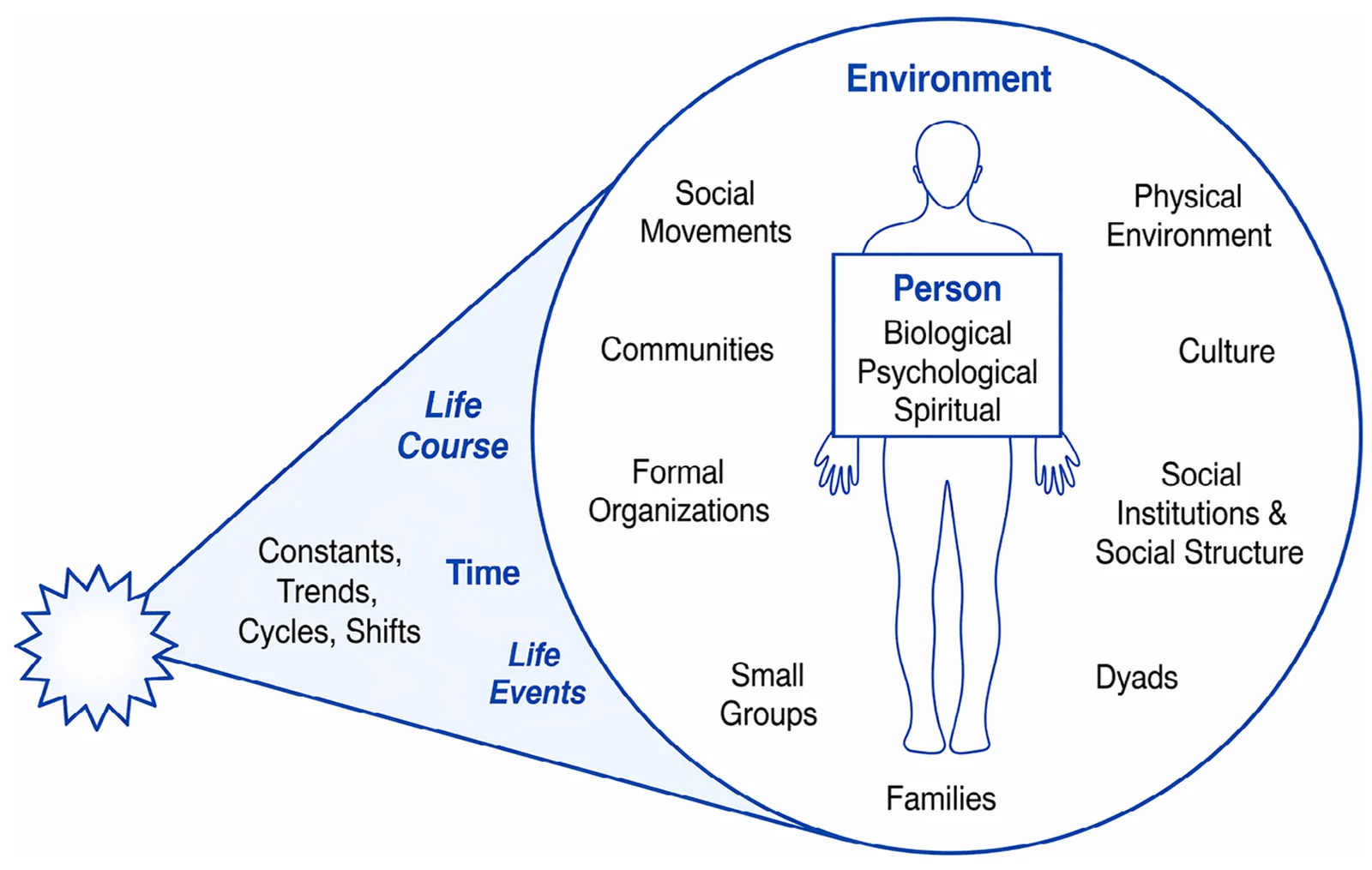
Life course theory/perspective. Elizabeth D. Hutchison, Virginia Commonwealth University Emerita and contributors. Dimensions of Human Behaviour: The Changing Life Course. Los Angeles: SAGE, 2015.

**TABLE 1:** Sample characteristics for the study sample of African American adults with criminal legal involvement and type 2 diabetes mellitus (*n* = 171).

	% or mean ± standard deviation
Age	57.2 ± 8.3

*Gender*	
Men	60.6%
Women	39.4%
Years of education	12.5 ± 2.6

*Income*	
<$25,000	87.6%
≥$25,000	12.4%

*Employment status*	
Not employed	81.4%
Employed	18.6%

*Marital status*	
Never married	55.3%
Married/separated/divorced/widowed	44.7%
BMI	35.5 ± 8.6
Duration of diabetes mellitus	17.8 ± 13.6
Comorbidity	4.2 ± 2.2

*Anti-diabetes medication*	
Pill or oral	44.3%
Insulin, shot or injectable	22.2%
Both	33.5%

**TABLE 2:** Summary of pre-incarceration variables and glycaemic control (*n* = 171).

	% or mean ± standard deviation
Haemoglobin A1c (Mean ± S.D)	8.0 ± 2.1

Emotional abuse	
Yes	17.5%

Physical abuse	
Yes	61.4%

Emotional neglect	
Yes	9.9%

Physical neglect	
Yes	17.5%

Sexual Abuse	
Yes	29.2%

Domestic violence	
Yes	47.4%

Witnessed violence	
Yes	62.6%

History of adverse neighbourhood conditions	
Yes	48.5%

History of substance abuse	
Yes	51.5%

History of mental health	
Yes	46.2%

History of incarceration	
Yes	43.3%

Discrimination	
Yes	71.4%

History of bully	
Yes	16.4%

Foster care	
Yes	8.2%

Conventional adverse childhood experience	
0	11.7%
1–3	41.5%
4+	46.8%

Expanded adverse childhood experience	
0	11.7%
1–2	47.4%
3+	40.9%

**TABLE 3:** Unadjusted, partially adjusted and stepwise linear regression models for the association between pre-incarceration social determinants of health and glycaemic control.

	Unadjusted β (95%CI)	Partially Adjusted β (95%CI)	Stepwise Regression β (95%CI)
*Pre-incarceration social determinants*			
Expanded ACE			
0	Reference	Reference	
1–2	−0.59 (−1.65, 0.46)	−0.69 (−1.86, 0.48)	
3+	−1.06 (−2.13, 0.01)	−1.07 (−2.34, 0.19)	−0.48 (−1.10, 0.14)

Conventional ACE			
0	Reference	Reference	
1–3	−0.05 (−1.12, 1.03)	−0.34 (−0.84, 1.52)	
4+	−0.52 (−1.58, 0.54)	0.05 (−1.20, 1.30)	

Locus of control	0.49 (−0.08, 1.06)	0.48 (−0.08, 1.05)	

*Demographics/clinical factors*			
Age			−0.05* **(−0.09, −0.01)**

Gender			
Men/Women			

Education			
Income			
Ref: <$25,000			
≥$25,000			

Employment status			
Not employed			Reference
Employed			−0.61 (−1.47, 0.25)

Marital status			
Ref: Never married			
Married/separated/divorced/widowed			
BMI			
Duration of diabetes			
Comorbidity			−0.22* **(−0.37, −0.08)**

Anti-diabetes medication			
Pill or oral			Reference
Insulin, shot or injectable			0.87*** **(0.07, 1.67)**
Both			1.30** **(0.60, 1.99)**

*Note*: Bold indicates statistical significance, CI = confidence interval. Empty boxes indicate variables dropped from the model based on stepwise regression.

*** *p* < 0.001.

** *p* < 0.01.

* *p* < 0.05.

## What’s new

- Pre-incarceration factors, including adverse childhood experiences and locus of control, were not associated with HbA1c in African Americans with criminal legal involvement and type 2 diabetes.
- Age, comorbidity burden and diabetes medication regimen were significantly associated with glycaemic control in this population.
- Findings suggest clinical factors may play a greater role than pre-incarceration social determinants in shaping glycaemic outcomes in justice-involved individuals.
